# Evaluation of the Association between Amount and Type of Milk Consumption and Periodontitis: Data from the Korea National Health and Nutrition Examination Survey (2016–2018)

**DOI:** 10.3390/nu15040914

**Published:** 2023-02-11

**Authors:** Eun Jeong Min, Siseong Jeong, Jun-Beom Park

**Affiliations:** 1 Department of Medical Life Science, College of Medicine, The Catholic University of Korea, Seoul 06591, Republic of Korea; 2 Department of Biomedicine & Health Sciences, The Catholic University Graduate School, Seoul 06591, Republic of Korea; 3 Department of Periodontics, College of Medicine, The Catholic University of Korea, Seoul 06591, Republic of Korea; 4 Dental Implantology, Graduate School of Clinical Dental Science, The Catholic University of Korea, Seoul 06591, Republic of Korea

**Keywords:** epidemiology, health surveys, milk, oral health

## Abstract

This study evaluated the association between the consumption of milk and having severe periodontitis. It is based on the information from the 2016–2018 Korea National Health and Nutrition Examination Survey. Severe periodontitis was characterized as a community periodontal index of code 4. A total of 18,034 individual respondents (7835 men and 10,199 women) without missing values were included in this study. Adjusted odds ratios and a 95% confidence interval of periodontitis in a multivariate logistic regression model for the amount of milk consumption were 0.774 [0.633–0.945] after the adjustment of confounding factors. This trend was maintained in a subgroup analysis of males with adjusted odds ratios, with a 95% confidence interval of 0.705 [0.538–0.924]. Overall, the findings showed a negative association between Korean adults’ milk consumption and the prevalence of severe periodontitis. Men with higher milk consumption were more likely to have a lower prevalence of severe periodontitis regardless of age, body mass index, smoking or drinking habits, education, income, region, and physical exercise, diabetes mellitus, hypertension, metabolic syndrome, white blood cell count and toothbrushing frequency. By contrast, in women, the amount of milk consumption was not significantly associated with severe periodontitis. The amount of milk consumed was discovered to be a potential risk indicator for severe periodontitis in men in this study.

## 1. Introduction

Our diet benefits from the nutrients that dairy products provide, such as energy, calcium, protein, and other micro- and macronutrients [1]. The intake of dairy foods, including milk, has been evaluated for local and systematic health. An anti-inflammatory impact was shown in prior research in both systemically healthy and metabolically disturbed participants [2]. The consumption of dairy products was associated with a lower incidence of type 2 diabetes and cardiovascular disease [3]. However, short-term low-fat milk consumption exhibited neither positive nor negative effects on the metabolic syndrome’s cardiometabolic risk variables in postmenopausal women with abdominal obesity [4]. In contrast, a prior study revealed that women who consumed more milk had a higher risk of fractures [5].

The effects of milk on oral health have been tested in previous studies [6,7,8,9,10]. Diet is reported as one of the major etiological factors for oral disease, including dental caries and enamel erosion [11]. The odds ratios of root caries occurrence for participants who did not drink milk every day were 1.69 (*p <* 0.05) [6]. Whole milk showed the lowest demineralization, followed by skim milk and then milk-based drinks, showing highest demineralization [7]. The regular consumption of milk and yogurt significantly reduced the abundance of these microbial taxa and also decreased the roughness of the enamel surface [8]. It was demonstrated that whole milk exposure resulted in lower bacterial counts than the milk-based drink, the milk-based drink with sucrose added to it, and the positive control of 20 percent sugar [7]. In contrast, another study indicated that drinking milk four or more days a week reduced the risk of dental caries with odds ratios of 0.34 [9]. More recently, the association between different beverages and oral health outcomes in aging population (older adults) was shown and it was found that periodontal disease was inversely related to milk [10]. This study’s objective was to evaluate, using nationally representative data, the association between milk consumption and periodontitis.

## 2. Materials and Methods

### 2.1. Overview of the Survey

The Institutional Review Board of Seoul, St Mary’s Hospital, College of Medicine, The Catholic University of Korea reviewed and approved the present work (KC21ZISE0940; approval date: 9 December 2021) and all methods were carried out in accordance with relevant guidelines and regulations. For this study, the Division of Chronic Disease Surveillance of the Korea Centers for Disease Control and Prevention and the Korean Ministry of Health and Welfare provided the data from the Korea National Health and Nutrition Examination Survey (KNHANES), which was conducted between 2016 and 2018 in Korea [12]. KNHANES is nationwide research on non-institutionalized civilians that used a rolling survey-sampling approach and a stratified, multi-stage probability sampling strategy [13]. The 2005 National Census Registry in Korea’s population and housing consensus, which takes into account factors including age, sex, and region, served as the basis for the sampling units [14]. Data from a total of 18,034 people (7835 males and 10,199 women) were used in this study, and only participants with complete data sets were included. The survey was divided into three sections: a nutrition survey, a health interview survey, and a health examination survey. Face-to-face interviews with standardized questionnaires were conducted by trained interviewers. A mobile examination center was used to conduct physical examinations, collect blood samples, and collect urine samples.

### 2.2. Demographic Variables

Demographic characteristics included sex, age, alcohol consumption, level of periodontitis, and smoking status. Consumption of milk was calculated based on the survey. Frequency, amount and type of milk were analyzed. Using the typical number of alcoholic beverages consumed and the frequency of alcohol intake, the amount of pure alcohol ingested (in grams per day) was estimated. The individuals were divided into four levels based on their scores on the alcohol use disorders identification test (AUDIT score; 0–7, 8–14, 15–19, 20) [15,16]. Heavy drinking was defined as consuming more than 60 g of pure alcohol per day for men and more than 40 g per day for women [17]. The low income category consisted of those whose household incomes fell into the lowest quartile, while the rest were classified as high income [18]. Whether the respondent had completed schooling beyond high school or had graduated from high school determined the respondent’s educational level. Smoking status was divided into three categories: present smokers, former smokers, and non-smokers. Physical exercise was defined as an average of days of walking, muscle-strengthening exercise or flexibility exercise at least three times a week for a minimum of 20 min [19]. With the aid of a systematic food frequency questionnaire, daily calcium intake was assessed [20]. In-person interviews were performed to gather information about the participant’s occupation and place of residence [20].

### 2.3. Anthropometric Evaluation

The Division of Chronic Disease Surveillance of the Korea Centers for Disease Control and Prevention and the Korean Ministry of Health and Welfare employed trained staff members to measure the participants. The subjects were wearing light indoor clothing and were not wearing shoes when their body weight and height were measured to the nearest 0.1 kg and 0.1 cm, respectively [21]. The narrowest part of the waist, which is located between the iliac crest and the lower border of the rib cage, was measured. The following formula was used to determine body mass index: weight/height^2^ (kg/m^2^). Systolic and diastolic blood pressure were measured on the right arm using a standard mercury sphygmomanometer (Baumanometer; W.A. Baum Co., Inc., Copiague, NY, USA). Systolic and diastolic blood pressure measurements were made twice at 5 min intervals, and the average values were used for the analysis.

### 2.4. Biochemical Analyses

Each participant’s antecubital vein was used to draw blood after they had fasted for more than eight hours in order to measure the levels of insulin, white blood cell count, fasting plasma glucose (FPG), total cholesterol (TC), high-density lipoprotein cholesterol (HDL-C), triglycerides (TG), and serum 25-hydroxyvitamin D. Blood samples were properly prepared, quickly chilled, and sent to the Central Testing Institute in cold storage (Seoul, Korea). In the course of the 24 h transportation period, blood samples were tested.

Using a 25-hydroxyvitamin D ^125^I RIA kit (DiaSorin, Stillwater, MN, USA), serum 25-hydroxyvitamin D levels were determined using a gamma counter (1470 Wizard; PerkinElmer, Wallac, Turku, Finland). A Hitachi Automatic Analyzer 7600 (Hitachi, Tokyo, Japan) was used to measure the levels of FPG, TC, HDL-C, and TG utilizing enzymatic techniques using commercially available kits (1470 Wizard, Perkin Elmer). Using a gamma counter and an immunoradiometric test kit (Biosource INS-IRMA kit, Biosource Europe SA, Nivelles, Belgium), the levels of insulin were measured. Laser flow cytometry (XE-2100D, Sysmex, Kobe, Japan) was used to calculate the white blood cell counts [14].

### 2.5. Description of Metabolic Syndrome, Diabetes and Hypertension

The American Heart Association/National Heart, Lung, and Blood Institute Scientific Statement criteria for Asians were used to establish metabolic syndrome [22]. Three or more of the following requirements must be met in order to be diagnosed with metabolic syndrome: waist circumference of less than 90 cm for men and 80 cm for women; fasting TG of less than 150 mg/dL; HDL-C of less than 40 mg/dL for men and 50 mg/dL for women; blood pressure of less than 130/85 mm Hg; and FBG of less than 100 mg/dL or current usage of anti-diabetes medication [12]. Diabetes was diagnosed when fasting blood sugar was >126 mg/dL or when the individual was using anti-diabetic medications at that time [23]. Systolic blood pressure of more than 160 mm Hg, diastolic blood pressure of more than 90 mm Hg, or current usage of systemic antihypertensive medications were all considered to be indicators of hypertension [24]. When hemoglobin fell below 12 g/dL in non-pregnant women, 11 g/dL in pregnant women, or 13 g/dL in men, anemia was identified [18].

### 2.6. Description of Periodontitis and Dental Caries

A CPI probe (PWHO, Osung MND, Seoul, Korea) with a 0.5 mm ball tip that adhered to WHO standards was employed. The mouth was divided into sextants and only when there were at least two teeth present that were not going to be extracted did a sextant examination take place. All remaining teeth in a sextant that qualified for examination were evaluated if no index teeth were present, and the highest score was recorded as the sextant’s score. The CPI score indicated the degree of periodontitis as follows: 0 indicates normal, 1 indicates gingival bleeding, 2 indicates calculus, 3 indicates moderate periodontitis or a shallow pocket with a depth of 3.5 to 5.5 mm, and 4 indicates severe periodontitis or a deep pocket with a depth of 5.5 mm or more [25]. The maximum CPI was chosen as a representative value, and if the representative value was greater than, or equal to, code 4 it was considered severe periodontitis and if the value was equal to or lower than 3 it was considered moderate periodontitis [12,26].

The measurements involved a probing force of about 20 g. Dentists with specialized training who finished the calibration process evaluated the participants’ periodontal health [12,26]. As part of the quality control, training was given to each examiner to reduce measurement errors of periodontal pocket depth during the examination. The number of teeth with dental caries was determined by adding the number of decayed teeth, the number of filled teeth, and the number of missing teeth.

### 2.7. Determination of Oral Health Behaviors

This study looked at when people used their toothbrushes and whether they used any other oral care products. We divided the total number of times per day that people brushed their teeth into daily frequency. Secondary oral care items included dental floss, mouthwash, an electric toothbrush, and an interdental brush [27]. Additionally, whether the subject had an oral examination within one year was evaluated. The self-reported oral health status was assessed, and it was divided into three categories: favorable, average, and problematic.

### 2.8. Analyses of Statistics

The statistical software package SAS version 9.4 for Windows, SAS Institute, Cary, NC, USA, was used to conduct the statistical analysis. Statistics were regarded as significant when two-sided *p* values were less than 0.05. Participants’ anthropometric, hematological, and demographic data are provided as means with standard errors to show whether they have severe periodontitis or not. The differences in the presence of severe periodontitis according to the variables were investigated using various statistical hypothesis testing methods. To test whether the population mean values of a continuous variable such as age for two groups are equal, we conducted Student’s t-test. For the comparison of population mean among more than three groups, we conducted one-way analysis of variance (ANOVA). Additionally, we conducted a chi-square test for the categorical variables, which compares the distribution of a variable in multiple groups. Logistic regression is well-known method for estimating the relationship between predictor variables and binary response variables. To access correlations between the level of milk consumption and the severe periodontitis, a multivariable logistic regression model was applied to the data while adjusting other confounding variables. From the regression results, odds ratios and 95% confidence intervals were calculated when potential confounders were taken into account.

## 3. Results

The characteristics of this present study are presented in Table 1. Age, waist circumference, systolic pressure, fasting glucose diabetes, hypertension, and metabolic syndrome were all greater in males with severe periodontitis when compared with males with moderate periodontitis (*p <* 0.05). The amount and type of milk consumption differed between the groups. In male participants, males with a high frequency of milk consumption were relatively higher in mild periodontitis (19.4%) than in severe periodontitis (12.3%). This was similar in the female group, showing that females with a high frequency of milk consumption were relatively higher in mild periodontitis (23.4%) than in severe periodontitis (19.3%). In male participants, males with high amounts of milk consumption were relatively higher in mild periodontitis (72.8%) than in severe periodontitis (54.2%). This was similar in females, and females with a high frequency of milk consumption were relatively higher in mild periodontitis (68.6%) than in severe periodontitis (57.9%). The majority of the participants consumed whole milk (47.3% in male and 42.3% in female), irrespective of whether they were male or female.

The relationship between oral health and severe periodontitis is shown in Table 2. There was a significantly higher number of teeth with dental caries experienced in males with severe periodontitis. A similar phenomenon was seen in females, with the number of teeth with dental caries experienced 7.7 ± 0.1 for mild periodontitis and 9.3 ± 0.3 for severe periodontitis, respectively (*p <* 0.05). The frequency of daily toothbrushing was lower in males with severe periodontitis when compared with participants with mild periodontitis (*p <* 0.05). The relative percentage of participants who did not use secondary oral products was 51.8% for males with mild periodontitis and 61.7% for severe periodontitis. The relative percentage of participants who did not use secondary oral products was lower for females with mild periodontitis when compared with females with severe periodontitis.

Table 3 displays the adjusted odds ratios and 95% confidence intervals for severe periodontitis in the multivariable logistic regression model for milk consumption frequency, categorized by <4 times/week versus ≥5 times/week. The association between frequency of milk consumption and severe periodontitis was not shown after adjustment with age, body mass index, smoking, drinking, education, income, region, and physical exercise, diabetes mellitus, hypertension, metabolic syndrome, white blood cell count, and toothbrushing frequency. This trend was maintained in the subgroup analysis of males and females.

Adjusted odds ratios, the 95% confidence interval of severe periodontitis in the multivariable logistic regression model for the amount of milk consumption categorized by (1) less than one cup/month versus (2) one cup or higher/month are shown in Table 4. There was negative association between the amount of milk consumption and severe periodontitis. This trend was maintained in the subgroup analysis of males. However, this trend was not maintained in the subgroup analysis of females. As we can observe, no significant association was noted once adjustment was carried out in Model 2, Model 3, and Model 4, and Model 5 (*p >* 0.05).

Table 5 contains the results of multivariate logistic analyses to study the relationship between the type of milk and severe periodontitis. It turned out that the type of milk did not have any significant association with severe periodontitis when adjustments were made (*p >* 0.05). Similarly, there was no association in male or female subgroups.

The subgroup analysis of oral health categorized by milk consumption frequency is shown in Table 6. The frequency of toothbrushing per day differed by the frequency of milk consumption in males and females (*p <* 0.05). The percentage of participants using secondary oral products differed by the frequency of milk consumption in males and females (*p <* 0.05). The percentage of participants with dental check-ups within a year differed by the frequency of milk consumption in the female group only (*p <* 0.05).

## 4. Discussion

The purpose of this study was to determine any links between milk consumption and severe periodontitis. The results presented decreased adjusted odds ratios of severe periodontitis with high amount of milk consumption in adults at a statistically significant level.

This study found that a high consumption of milk may have beneficial effects on oral health, leading to fewer events of severe periodontitis. Milk-derived beverages are considered to be beneficial not only to health but also to dental tissue [10]. The effects of milk on oral health have been tested in different geographical regions [28,29]. The prevalence of periodontal diseases and its relationship to various risk factors was investigated among the adult population of Egypt, and the results showed that calculus was negatively correlated with milk, brushing frequency, socioeconomic status and education level [28]. An evaluation of the factors that contribute to periodontitis in a rural Indian population and multivariate analysis which was performed to predict periodontitis showed that not drinking milk was shown to be one of six risk factors with adjusted odds ratios = 1.7 and 95% confidence interval = 1.29–2.24 [29]. A previous study looked at the connection between periodontal health and the consumption of dairy products such as milk, cheese, and meals high in lactic acid (yogurt and lactic acid drinks) [30]. In multivariable logistic regression analysis, subjects eating ≥ 55 g dairy products including milk (lactic acid foods) per day had a significantly lower prevalence of deep probing depth and severe clinical attachment loss compared to those not eating these foods, after adjusting for confounding variables; the odds ratios for generalized deep probing depth and severe clinical attachment loss were 0.40 (95% confidence interval: 0.23 to 0.70) and 0.50 (95% confidence interval: 0.29 to 0.87), respectively [30]. It is interesting to note that the prevalence of periodontal disease was 0.83 times lower when drinking coffee with cream than when drinking black coffee, and it has been proposed that doing so may help to prevent the condition [31]. Conversely, in another report, periodontal disease progression developed over six years in a non-drinking group of participants compared with those who drank milk every day was 95.7% and 95.8%, respectively [6].

The differences between males and females regarding milk consumption have been analyzed. A prior study using information from the FoodNet Population Surveys found no sex differences in milk intake [32]. The consumption of milk produced different responses between females and males [33]. Compared to other groups, women who drank milk more than once a day were younger and engaged in less physical activity, but men did not exhibit this difference [33]. In this study, Korean male adults with severe periodontitis consumed considerably less milk than healthy controls. Regardless of age, body mass index, smoking, drinking, education, income, region, physical activity, diabetes mellitus, hypertension, metabolic syndrome, white blood cell count, and frequency of brushing their teeth, men who consumed more milk were more likely to have a lower prevalence of severe periodontitis. Reduced-fat milk intake was found to have a lower mortality risk than that of whole milk in studies that looked at whole milk, reduced-fat milk, and skim milk consumption among men and women with cardiovascular disease [34]. This study concluded that the type of milk did not have any significant association with severe periodontitis.

Milk consumption may lead to the control of inflammation. In a previous study, the anti-inflammatory effects on oral fibroblasts and oral epithelial cells as well as the cells of the oral cavity were examined [35]. Milk may have a protective effect on the oral cavity by modulating the macrophage-based innate immune system, according to a previous study that found that milk led macrophages to polarize from a pro-inflammatory M1 phenotype towards a pro-resolving M2 phenotype [36]. Transforming growth factor-β, which maintains mucosal homeostasis, is abundant in milk, and gingival fibroblasts respond to milk by upregulating the expression of target genes for transforming growth factor-β [37]. Clinical studies looking at inflammatory markers in relation to dairy product consumption revealed anti-inflammatory activity in humans [38]. Both healthy patients and those with metabolic abnormalities showed a sizable anti-inflammatory impact [2]. It has been suggested that casein, calcium, and lactose in milk can induce intestinal alkaline phosphatase, a powerful endogenous anti-inflammatory enzyme, which can then dephosphorylate and detoxify pro-inflammatory microbial components [39]. Additionally, it was proposed that the milk fat globule’s surface carbohydrate moieties and antimicrobial peptides may play a crucial role in determining the composition of the gut microbial population, which in turn may strengthen defenses against inflammatory and immunological disorders [40]. Increased oral health can be obtained by the consumption of fortified milk. Probiotic dairy products are reported to be promising alternatives for improving oral health [41]. Probiotics can change an unhealthy bacterial environment into one that is healthy, and a prior study found that probiotic milk drinks have a positive impact on periodontopathic bacteria in dental biofilms [42]. Oral clearance rates for fruit drinks and coffee were found to be identical at 15 min, while those for sweetened milk were found to be the least at 6.5 min [11]. Daily or triweekly probiotic milk consumption can only somewhat prevent new caries, but can significantly reverse existing carious lesions, suggesting that reversing carious lesions only requires a daily or triweekly dosing interval [43]. There was a reduction of caries in permanent teeth and primary teeth with the use of fluoridated milk, with a higher reduction in primary teeth, and it was proposed that milk could be a reasonably affordable means of delivering fluoride to prevent dental cavities [44]. The mineral density of synthetic proximal carious lesions in situ was increased by fluoridated milk [45]. The effect of a fluoridated milk-based beverage on an in vitro root caries experimental model was evaluated with the aim of preventing caries in older persons [46]. Additionally, calcium/vitamin D milk was regarded as a nutritious and secure diet for expectant mothers [47].

This study had several limitations. First, because of its cross-sectional observational methodology, the current study is unable to definitively establish the precise causal link between milk consumption and severe periodontitis [48]. To establish the specific impact of milk consumption on periodontitis, additional longitudinal cohort studies are required. Second, the credibility of the study may be affected by the possibility that milk volume content varies between cups of different sizes [49]. The fact that this study only used recollection to determine people’s eating habits is another drawback [50]. The KNHANES, a nationally representative sample whose participants were chosen using a multi-stage clustered probability design, provided the data for this investigation [51]. Multiple logistic regression models were used to assess the relationship between milk consumption and severe periodontitis after controlling for confounding variables [52]. Additionally, the findings of this study can be regarded as trustworthy and applicable to a broad audience.

Overall, the findings showed a link between Korean adults’ milk consumption and their chance of developing severe periodontitis. Men with higher milk consumption were more likely to have a lower prevalence of severe periodontitis regardless of their age, body mass index, smoking, drinking, education, income, region, and physical exercise, diabetes mellitus, hypertension, metabolic syndrome, white blood cell count and toothbrushing frequency. By contrast, in women, the amount of milk consumption was not significantly associated with severe periodontitis. The amount of milk consumed was discovered to be a potential risk indicator for severe periodontitis in men in this study. Additionally, the frequency of tooth brushing and the percentage of participants using secondary oral products differed by the frequency of milk consumption both in males and females, respectively.

## Figures and Tables

**Table 1:** Characteristics of the study population.

Characteristics	Periodontitis					
	Moderate	Severe	*p*-Value	Moderate	Severe	*p*-Value
	Male (*n* = 7835)			Female (*n* = 10,199)		
Age (years)	43.5 ± 0.3	58.2 ± 0.5	<0.0001	46.0 ± 0.3	62.1 ± 0.6	<0.0001
Height (cm)	171.5 ± 0.1	168.0 ± 0.2	<0.0001	157.8 ± 0.1	153.6 ± 0.3	<0.0001
Weight (kg)	71.8 ± 0.2	69.1 ± 0.4	<0.0001	57.6 ± 0.1	57.1 ± 0.4	0.253
Body mass index (kg/m^2^)	24.4 ± 0.1	24.4 ± 0.1	0.6855	23.2 ± 0.1	24.2 ± 0.1	<0.0001
Waist circumference (cm)	84.8 ± 0.2	86.3 ± 0.3	<0.0001	77.6 ± 0.2	81.7 ± 0.4	<0.0001
Systolic pressure (mmHg)	118.8 ± 0.3	122.2 ± 0.6	<0.0001	113.4 ± 0.3	123.8 ± 0.8	<0.0001
Diastolic pressure (mmHg)	77.7 ± 0.2	77.2 ± 0.4	0.2608	72.4 ± 0.2	72.9 ± 0.5	0.251
Fasting glucose (mmol/dL)	100.1 ± 0.4	108.5 ± 1.4	<0.0001	96.1 ± 0.3	106.2 ± 1.4	<0.0001
HbA1c (%)	5.7 ± 0.0	6.1 ± 0.0	<0.0001	5.7 ± 0.0	6.1 ± 0.0	<0.0001
Total cholesterol (mmol/dL)	187.4 ± 0.6	188.9 ± 1.4	0.3147	188.1 ± 0.5	191.2 ± 1.7	0.073
High density lipoprotein (mg/dL)	47.5 ± 0.2	46.0 ± 0.4	0.0011	54.9 ± 0.2	51.3 ± 0.6	<0.0001
Triglycerides (mmol/dL)	161.7 ± 2.4	172.2 ± 6.2	0.114	109.3 ± 1.0	130.5 ± 4.3	<0.0001
Creatinine (mg/dL)	0.98 ± 0.01	1.01 ± 0.03	0.3475	0.72 ± 0.00	0.74 ± 0.01	0.011
WBC count × 10^9^/L	6.7 ± 0.0	7.1 ± 0.1	<0.0001	6.0 ± 0.0	6.3 ± 0.1	0.0001
Insulin (μU/mL)	8.8 ± 0.2	7.7 ± 0.3	0.0028	8.1 ± 0.2	10.2 ± 0.7	0.008
Vitamin D (ng/mL)	16.9 ± 0.2	18.2 ± 0.6	0.0269	15.5 ± 0.2	16.6 ± 0.6	0.072
Calcium intake (mg/day)	536.8 ± 5.4	467.7 ± 11.4	<0.0001	475.2 ± 4.1	429.5 ± 12.4	0.0004
Hypertension						
No	73.8	9.2	<0.0001	76.5	5.3	<0.0001
Yes	12.9	4.1		14.8	3.3	
Diabetes mellitus						
Normal	57.4	6.1	<0.0001	70.7	4.2	<0.0001
Impaired fasting glucose	21.8	3.9		15.2	2.2	
Diabetes mellitus	7.9	2.9		6.1	1.6	
Anemia						
No	84.4	12.5	0.0001	81.5	7.3	0.2793
Yes	2.5	0.7		10.2	1.1	
Metabolic syndrome						
No	57.1	8.9	<0.0001	60.5	6.0	<0.0001
Yes	27.5	6.5		28.1	5.4	
Housing (region)						
City(-si)	41.9	5.3	0.0001	45.5	3.7	0.001
Province(-do)	44.5	8.3		45.5	5.3	
Income level						
High	28.2	3.3	<0.0001	28.0	1.6	<0.0001
Low	58.2	10.3		63.0	7.3	
Education						
Lower than high school graduate	14.6	5.3	<0.0001	25.9	5.5	<0.0001
High school graduate or higher	72.1	8.0		65.4	3.2	
Occupation classification						
Manager	15.3	1.5	<0.0001	12.4	0.4	<0.0001
Officer worker	12.5	0.9		8.4	0.2	
Service	11.1	1.3		13.1	1.2	
Agricultural, forestry and fishery workers	3.5	1.3		2.1	0.5	
Craftsman	16.8	2.9		2.3	0.2	
Simple labor	6.2	1.2		7.8	1.3	
Unemployed	21.5	4.2		45.2	4.9	
**AUDIT score**						
0–7	43.4	6.3	0.0069	79.6	6.6	0.006
8–14	26.3	3.0		9.7	0.3	
15–19	10.2	2.0		2.0	0.1	
≥20	7.3	1.5		1.7	0.0	
**Heavy drinking**						
No	26.8	5.3	<0.0001	62.5	7.0	<0.0001
Yes	59.9	8.0		28.9	1.6	
**Frequency of drinking in one year**						
Less than once a month	22.4	4.1	<0.0001	51.6	6.1	<0.0001
1 to 4 times a month	35.4	4.0		29.9	1.8	
Two or more times a week	28.9	5.2		9.9	0.7	
**Smoking**						
Nonsmoker	23.1	1.6	<0.0001	81.4	7.6	0.356
Ex-smoker	30.0	5.8		5.3	0.4	
Current smoker	33.6	6.0		4.7	0.5	
**Regular physical exercise (average number of days per week)**	3.2 ± 0.0	3.0 ± 0.1	0.0009	2.9 ± 0.0	2.7 ± 0.1	0.0003
**Frequency of milk consumption**						
Low	72.0	9.3	0.0014	72.2	4.6	0.078
high	17.3	1.3		22.1	1.1	
**Amount of milk consumption**						
Low	24.3	4.9	<0.0001	29.6	2.4	0.0003
high	65.1	5.8		64.7	3.3	
**Type of milk**						
Whole milk	47.3	4.4	<0.0001	42.3	2.3	0.001
Low-fat milk	9.2	1.2		17.6	0.8	
Similar (whole/low-fat)	11.3	0.6		10.0	0.5	
Not applicable (<once/month)	21.6	4.5		24.3	2.1	

The data were presented as the mean ± standard deviation for continuous variables or the percentage for categorical variables.

**Table 2:** The relationship between oral health/oral habit and periodontitis.

Characteristics	Periodontitis					
	Moderate	Severe	*p*-Value	Moderate	Severe	*p*-Value
	Male			Female		
Number of teeth with dental caries	0.8 ± 0.0	0.8 ± 0.1	0.8414	0.6 ± 0.0	0.6 ± 0.1	0.7352
Number of teeth experienced with dental caries	6.2 ± 0.1	6.5 ± 0.2	0.1951	7.7 ± 0.1	9.3 ± 0.3	<0.0001
Toothbrushing yesterday						
No	1.1	0.6	<0.0001	0.8	0.3	<0.0001
Yes	85.6	12.7		90.6	8.2	
Frequency of daily toothbrushing	2.5 ± 0.0	2.3 ± 0.0	<0.0001	2.7 ± 0.0	2.4 ± 0.0	<0.0001
Oral examination within a year						
No	60.5	9.9	0.025	62.9	6.5	0.0003
Yes	26.2	3.4		28.6	2.1	
Self-reported oral status						
Favorable	14.7	1.1	<0.0001	12.7	0.9	<0.0001
Average	35.8	3.4		41.7	3.0	
Problematic	35.9	9.1		36.6	5.1	
Use of secondary oral products						
No	44.9	8.2	0.0017	38.0	4.9	<0.0001
Yes	41.8	5.1		53.4	3.6	

**Table 3:** Adjusted odds ratios, 95% confidence interval, and *p*-value of severe periodontitis in multivariable logistic regression model for frequency of milk consumption (<4 times/week versus ≥ 5 times/week).

Model	Total		Male		Female	
	Adjusted Odds Ratios (95% Confidence Interval)	*p*-Value	Adjusted Odds Ratios (95% Confidence Interval)	*p*-Value	Adjusted Odds Ratios (95% Confidence Interval)	*p*-Value
Model 1	0.628 (0.496, 0.794)	0.000	0.589 (0.424, 0.819)	0.002	0.759 (0.558, 1.032)	0.079
Model 2	0.951 (0.709, 1.276)	0.738	0.957 (0.618, 1.481)	0.842	0.996 (0.675, 1.471)	0.986

Model 1 was unadjusted. Model 2 was adjusted for age, body mass index, smoking, drinking, education, income, region, and physical exercise, diabetes mellitus, hypertension, metabolic syndrome, white blood cell count, and toothbrushing frequency.

**Table 4:** Adjusted odds ratios, 95% confidence interval, and *p*-value of severe periodontitis in multivariable logistic regression model for amount of milk consumption (< one cup/month versus ≥ one cup/month).

Model	Total		Male		Female	
	Adjusted Odds Ratios (95% Confidence Interval)	*p*-Value	Adjusted Odds Ratios (95% Confidence Interval)	*p*-Value	Adjusted Odds Ratios (95% Confidence Interval)	*p*-Value
Model 1	0.520 (0.443, 0.610)	<0.0001	0.443 (0.356, 0.551)	<0.0001	0.635 (0.496, 0.813)	0.0003
Model 2	0.774 (0.633, 0.945)	0.012	0.705 (0.538, 0.924)	0.011	0.918 (0.674, 1.250)	0.586

Model 1 was unadjusted. Model 2 was adjusted for age, body mass index, smoking, drinking, education, income, region, and physical exercise, diabetes mellitus, hypertension, metabolic syndrome, white blood cell count, and toothbrushing frequency.

**Table 5:** Adjusted odds ratios, 95% confidence interval, and *p*-value of severe periodontitis in multivariable logistic regression models for consumption of types of milk (whole milk and low-fat milk) when compared with consumption of similar proportions of both types.

Model	Type of Milk	Total		Male		Female	
		Adjusted Odds Ratios (95% Confidence Interval)	*p*-Value	Adjusted Odds Ratios (95% Confidence Interval)	*p*-Value	Adjusted Odds Ratios (95% Confidence Interval)	*p*-Value
Model 1	Whole	1.493 (1.089, 2.048)	0.013	1.873 (1.134, 3.093)	0.014	1.071 (0.681, 1.683)	0.767
	Low-fat	1.474 (1.016, 2.139)	0.041	2.554 (1.409, 4.628)	0.002	0.905 (0.527, 1.556)	0.719
Model 2	Whole	1.104 (0.736, 1.655)	0.633	1.190 (0.633, 2.239)	0.589	1.013 (0.593, 1.729)	0.962
	Low-fat	0.791 (0.482, 1.300)	0.355	1.146 (0.541, 2.425)	0.722	0.520 (0.269, 1.005)	0.052

Model 1 was unadjusted. Model 2 was adjusted for age, body mass index, smoking, drinking, education, income, region, and physical exercise, diabetes mellitus, hypertension, metabolic syndrome, white blood cell count, and toothbrushing frequency.

**Table 6:** Subgroup analysis of oral health and oral habits categorized by milk consumption frequency.

Sex	Variables		Milk Consumption Frequency (%)									
			None	1 Per Month	2–3 Per Month	1 Per Week	2–3 Per Week	4–6 Per Week	1 Per Day	2 Per Day	3 Per Day	*p*-Value
Male	Frequency of toothbrushing per day	≤1	5.35	1.43	1.83	2.19	3.09	0.86	1.35	0.22	0.02	0.015
		2	9.56	3.27	4.13	4.97	8.48	2.07	3.54	0.69	0.07	
		≥3	11.46	3.77	4.92	5.80	11.09	3.28	5.39	0.82	0.34	
	Use of secondary oral product	No	14.30	4.43	5.99	5.68	11.17	2.88	4.83	0.65	0.27	0.018
		Yes	12.03	4.21	4.87	7.12	11.62	3.33	5.44	0.97	0.18	
	Dental check-up within a year	No	18.78	5.98	7.54	8.90	15.68	4.20	6.98	1.02	0.40	0.714
		Yes	7.56	2.65	3.33	3.90	7.11	2.01	3.29	0.60	0.06	
	Self-reported oral status	Favorable	3.75	1.28	1.92	2.21	3.46	1.01	1.87	0.30	0.06	0.507
		Average	10.28	3.35	4.68	5.80	9.60	2.50	4.06	0.77	0.09	
		Problematic	12.10	3.86	4.33	5.14	9.61	2.72	4.41	0.53	0.32	
Female	Frequency of toothbrushing per day	≤1	2.82	0.60	0.89	0.88	1.70	0.43	1.17	0.23	0.01	0.007
		2	8.63	2.49	3.22	3.51	6.87	1.50	4.00	0.57	0.06	
		≥3	15.02	3.98	5.76	6.76	13.69	3.94	10.11	0.96	0.19	
	Use of secondary oral product	No	11.39	3.01	3.62	3.83	8.05	2.16	4.98	0.55	0.09	0.001
		Yes	15.11	4.11	6.16	7.42	14.23	3.75	10.24	1.11	0.18	
	Dental check-up within a year	No	18.30	4.81	6.17	7.20	14.48	3.65	9.87	1.11	0.09	0.049
		Yes	8.20	2.32	3.62	4.05	7.78	2.25	5.35	0.56	0.18	
	Self-reported oral status	Favorable	2.92	0.84	1.10	1.65	3.05	0.83	2.34	0.21	0.06	0.053
		Average	11.81	3.57	4.85	5.41	10.62	3.07	7.26	0.96	0.12	
		Problematic	11.71	2.73	3.79	4.24	8.57	1.90	5.67	0.60	0.10	

## Data Availability

This publication contains all of the data that were created or examined during this study.
